# Language Effects in Trilinguals: An ERP Study

**DOI:** 10.3389/fpsyg.2012.00402

**Published:** 2012-10-22

**Authors:** Xavier Aparicio, Katherine J. Midgley, Phillip J. Holcomb, He Pu, Jean-Marc Lavaur, Jonathan Grainger

**Affiliations:** ^1^Université Montpellier Sud de FranceMontpellier, France; ^2^Tufts UniversityMedford, USA; ^3^Aix-Marseille UniversityMarseille, France

**Keywords:** language effects, trilingualism, visual word recognition, N400

## Abstract

Event-related potentials were recorded during the visual presentation of words in the three languages of French-English-Spanish trilinguals. Participants monitored a mixed list of unrelated non-cognate words in the three languages while performing a semantic categorization task. Words in L1 generated earlier N400 peak amplitudes than both L2 and L3 words, which peaked together. On the other hand, L2 and L3 words did differ significantly in terms of N400 amplitude, with L3 words generating greater mean amplitudes compared with L2 words. We interpret the effects of peak N400 latency as reflecting the special status of the L1 relative to later acquired languages, rather than proficiency in that language *per se*. On the other hand, the mean amplitude difference between L2 and L3 is thought to reflect different levels of fluency in these two languages.

## Introduction

The human ability to understand and speak more than one language has become a topic of central importance in contemporary cognitive psychology, perhaps in part due to the acknowledgment that in today’s world, multilingualism is the norm rather than the exception. The topic also likely attracts attention because of the interesting questions that arise when thinking about issues related to how information about the different languages is represented in the multilingual brain, and how access to language-specific information is controlled during language production and comprehension. However, while the number of studies investigating bilingualism has indeed shown a sharp increase in recent years, studies of those speaking more than two languages are still rather sparse. Yet the study of trilinguals not only raises some important questions in its own right (see e.g., Van Hell and Dijkstra, 2002; Lemhöfer et al., 2004, for studies of cognate effects in trilinguals), but also provides possible ways to tackle basic questions about bilingualism and second language acquisition in the same manner that studying bilingualism can shed light on language processing in general. In particular, having a third language should help disentangle issues of how age-of-acquisition, order-of-acquisition, and fluency might differentially affect non-native language use. In order to make some further progress in this direction, the present study examines event-related potentials (ERPs) generated by words in the different languages of trilingual persons performing a semantic categorization task.

The starting point of the present work is Midgley et al.’s (2009) ERP investigation of visual word recognition in bilinguals. Midgley et al. compared the ERPs generated by L1 and L2 words in bilingual participants with different levels of proficiency in their L2^1^. In Experiment 1, they tested beginning English-French bilinguals (American learners of French) in a silent reading task where participants had to indicate whether words belonged to a given semantic category or not. ERPs to L1 and L2 words differed in two notable ways. L1 words generated more negative-going waveforms than L2 words on the N400 component at posterior sites, and in anterior sites there was a clear delay in peak N400 latency for L2 words. In Experiment 2, Midgley et al. found a similar pattern of ERPs to L1 and L2 words with beginning French-English bilinguals (French learners of English), clearly indicating that the effect is due to language dominance and not language *per se* (i.e., which specific language is L1 or L2). However, in their third experiment with more balanced French-English bilinguals, they no longer saw a difference in N400 amplitude at posterior sites, but continued to, see the latency shift in anterior sites. This suggests that the anterior shift in N400 peak latency could be related to fundamental differences in processing L1 compared with later acquired languages independently of proficiency.

The impact of this hypothesized special status of the L1 has been highlighted in theoretical accounts of second language acquisition (e.g., Hernandez and Li, 2007). The key notion here is that in multilingual persons that have first learned L1 before learning other languages, then L1 acquisition is qualitatively different from the acquisition of a later learned L2 and any subsequently acquired languages. This point was made indirectly by Hernandez et al. (2005) when they drew a clear distinction between early and late bilingualism. These authors argued that late learners of an L2 use a more “parasitic” approach to language acquisition in general, and vocabulary acquisition in particular (see Grainger et al., 2010, for similar arguments pitched within the framework of a developmental version of the bilingual interactive-activation (BIA) model – Grainger and Dijkstra, 1992; van Heuven et al., 1998). The term “parasitic,” used by Hernandez et al. (2005), refers to the role of translation equivalents in L1 in order to access meaning from L2 words during the initial phase of L2 word learning. This is clearly a very different mechanism for learning the meaning of new words compared with L1 word learning, and is thought to impact on the nature of semantic representations and their connectivity with word form representations (see Grainger et al., 2010, for a specific proposal). Thus, the qualitatively different nature of vocabulary learning in L2 is hypothesized to result in a different lexical organization compared with the learning of words in L1. Note that this does not imply that late L2 learners cannot attain L1-like competence. The hypothesis is that since the acquisition process is different, this will have a fairly long-lasting impact on how the L2 is processed during comprehension and production.

As a further test of this hypothesis, in the present study we compared the processing of words in L1 with words in L2 and L3 in trilingual participants, where L2 and L3 are both later acquired languages. Here, the hypothesis under test is that if the L1 has a special status compared with later acquired languages, then this should be revealed in data where L2 and L3 pattern together and are both different from L1^2^. More precisely, we should observe an earlier peak N400 latency for L1 words with respect to both L2 and L3 words, which themselves should show similar peak latencies. On the other hand, any observed differences in the processing of L2 and L3 words could be more likely attributed to differences in proficiency level in these two languages, at least for the specific population of trilinguals tested in the present study.

In the present study we provide an additional test of the hypothesis that the earlier peak N400 latency to L1 words compared with L2 words seen in the Midgley et al. (2009) study reflects a possible special status of the L1, such as described by Hernandez et al. (2005) and Grainger et al. (2010). Furthermore, Midgley et al. hypothesized that differences in N400 amplitude seen in posterior sites might be more a reflection of differences in fluency between the L1 and the L2, since these effects were found to be reduced with higher levels of proficiency in the L2. That is, N400 amplitude was more negative-going, and hence closer to the N400 generated by L1 words, in the participants with a higher proficiency in L2. This would suggest that we ought to observe more negative-going N400 amplitudes in L2 than L3 in the present study, given the different levels of fluency in these two languages.

Summing up, in the present study ERPs were recorded to words in L1, L2, and L3 of French-English-Spanish trilinguals of differing L2 and L3 proficiency during a silent reading task. Our participants were all relatively late learners of their L2 and L3. They also started learning their L2 before the L3, and a number of measures indicated that their proficiency was higher in L2 than in L3 (see Tables 1 and 2). If the latency shift in anterior negativity seen in the Midgley et al. (2009) study is due to the special status of the L1, then we expect to, see a similar latency shift with respect to both L2 and L3, which should have peak N400 latencies that are delayed with respect to L1. On the other hand, if the latency shift is due to differences in proficiency level, then one would expect to, see graded effects across the three languages. However, differences in N400 amplitude generated by L2 and L3 words might be expected on the basis of differing levels of proficiency in these languages, with L2 generating a more negative N400 than L3.

**Table 1:** **Summary of participants’ language skills and relative use of each language**.

Percentage of daily use			Speaking skills			Comprehension skills			Reading frequency		
L1	L2	L3	L1	L2	L3	L1	L2	L3	L1	L2	L3
59% (20.5)	23% (13.3)	18% (9.8)	7.0 (0.0)	5.6 (1.1)	5.4 (1.3)	7.0 (0.0)	5.6 (1.0)	5.4 (0.9)	6.4 (1.0)	5.1 (1.2)	4.4 (1.5)

*Speaking and comprehension skills as well as reading frequency are evaluated on a seven-point scale (1 = poor/rarely, 7 = excellent/often)*.

*Mean across participants, standard deviations in parentheses*.

**Table 2:** **Summary of participants’ estimated age of onset of acquisition (AoOA) of L2 and L3 and post-test translation scores for L1–L2 and L1–L3**.

AoOA (years)		Post-test translation score	
L2	L3	L1 → L2	L1 → L3
10 (1.5)	13 (1.1)	88% (0.10)	72% (0.12)

*Mean across participants, standard deviations in parentheses*.

## Materials and Methods

### Participants

Eighteen undergraduate volunteers (15 women) from the University of Provence were recruited and paid for their participation. All were right-handed native speakers of French, and reported normal or corrected to normal vision and no history of neurological insult or language disability. Participants were enrolled in their third year of foreign language studies in English and Spanish. French was reported to be the first language learned by all participants (L1), English the second language (L2), and Spanish the third (L3). L2 and L3 were both learned in the classroom, and in addition to classroom learning participants reported having spent several weeks in immersion in English and Spanish speaking countries.

A questionnaire and translation task was used to evaluate participants’ fluency in L2 and L3. They were asked to estimate the percentage of daily use of each language. They also evaluated their language skills in L1, L2, and L3 on a seven-point Likert scale, as well as how often they read in each language (very rarely – very often, see Table 1). Participants rated their percentage of daily use to be higher in L1 than in L2 [*t*(17) = 12.9, *p* < 0.001], and higher in L2 than in L3 [*t*(17) = 2.1, *p* = 0.05], and they rated their frequency of reading to be higher in L1 than in L2 [*t*(17) = 4.8, *p* < 0.001], and higher in L2 than in L3 [*t*(17) = 2.4, *p* < 0.05]. Participants rated their speaking skills to be higher in L1 than in L2 [*t*(17) = 5.4, *p* < 0.001], but not significantly different in L2 and L3 [*t*(17) = 0.5], and similarly rated their comprehension skills to be higher in L1 than in L2 [*t*(17) = 6.2, *p* < 0.001], but not significantly different in L2 and L3 [*t*(17) = 0.5]. Participants were also asked to report the estimated age of onset of acquisition (AoOA) of each language. They estimated that L2 acquisition was initiated significantly earlier than L3 [*t*(17) = 86.1, *p* < 0.001]. Finally, a test of their translation abilities from L1 into L2 and from L1 into L3 was administered after the ERP session (see Table 2). In this post-test translation task participants were asked to translate a list of 70 L1 words into L2 and L3 in order to assess their knowledge of the critical items used in the ERP experiment and provide a measure of L2 and L3 vocabulary size. Stimuli used in the post-test were a subset of the critical words used in the experiment. The average frequency (occurrences per million – OPM) of these words in L1 was 95 OPM, and the corresponding frequencies of the translations in L2 and in L3 were respectively of 91 OPM and 93 OPM. Half of the participants generated the L2 translations first then the L3 translations, and the remaining participants did the translation in the opposite order. Participants could take their time, and on average they took approximately half an hour to complete the translation test. Performance on this task ranged from 70 to 100% correct in L2 (*M* = 88%), and from 53 to 96% correct in L3 (*M* = 72%). Performance was significantly better in L2 than L3 [*t*(17) = 17.9, *p* < 0.01].

### Stimuli

The French, English, and Spanish stimuli were selected from a trilingual database (Laxén et al., 2008) containing approximately 1500 translation equivalents (4500 words) from French, English, and Spanish ranked according to their degree of overlap in orthography, phonology, and semantics. The selected stimuli were 282 words that were non-cognates in French, English, and Spanish, with 94 words in each language. The words were selected to be as language-specific as possible, thus excluding identical cognates, close cognates (differing by a single letter substitution, deletion, or insertion), and interlingual homographs, in order to minimize confusion over which language was being presented. None of these words was a translation of another word in the list. The words were between three and eight letters in length, with a mean word frequency in French of 69 OPM (based on the Lexique 3 database), 66 OPM in English (based on the CELEX database), and a mean word frequency in Spanish of 66 OPM (based on the Lexesp database). We also selected 72 non-cognate animal words (24 in each language) for the purposes of the semantic categorization task, plus 270 filler words (90 in each language) that varied in cognate status.

All words were presented in lower case letters in 20 point Verdana font which resulted in a word height of 2.2 cm and a range of word length between 4.5 and 8.0 cm, which at a viewing distance of approximately 1.5 m resulted in word lengths ranging from 1.7° to 3.1° of visual angle. Words were centered on the screen and presented in white on a black background. No more than three words of the same language were presented in a row. On each trial, participants were presented with a single word and had to decide if the item referred to an animal in French, English, or Spanish. If an item referred to an animal (12% of trials), the participants were instructed to press a button on a response box as quickly as possible. For all other items, the critical items, and filler items, no overt response was expected. This task leaves critical items free of artifact from overt responding while requiring silent reading for meaning of each item.

### Procedure

**Figure 1:**
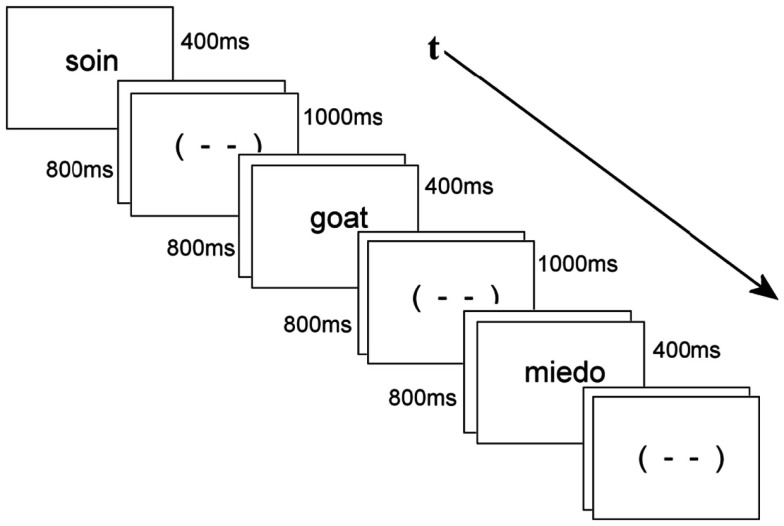
**Example of a sequence of stimuli, here an L1 word followed by an L2 animal probe followed by an L3 word – all separated by blink stimuli**.

Participants were tested in a sound attenuated room while seated in a comfortable armchair approximately 1.5 m from a computer monitor. Stimuli were displayed at the center of the monitor as white lower case letters on a black background. Each trial consisted of a stimulus word presented for 400 ms, that was followed by an 800 ms blank screen, a blink signal (- -) for 1000 ms (signaling it was permissible to blink or move one’s eyes), and a final blank screen lasting 500 ms. The stimulus word from the next trial immediately followed the final blank screen (see Figure 1 below). Order of presentation was pseudo-random. Participants pressed a button on a response box resting in their lap whenever a probe item (i.e., an animal name) appeared in any of the three languages. Participants were asked not to move or blink except when the blink signal “(- -)” appeared on the screen to reduce rejection of data due to artifact. A training session of 30 items proceeded the ERP session. None of the training items appeared in the main experiment. Altogether, the experiment lasted about 30 min including four breaks. At the end of the recording session participants were presented with 70 of the L1 words that they had already seen in the main experiment, and were asked to translate these into both L2 and L3.

### EEG recording procedure

**Figure 2:**
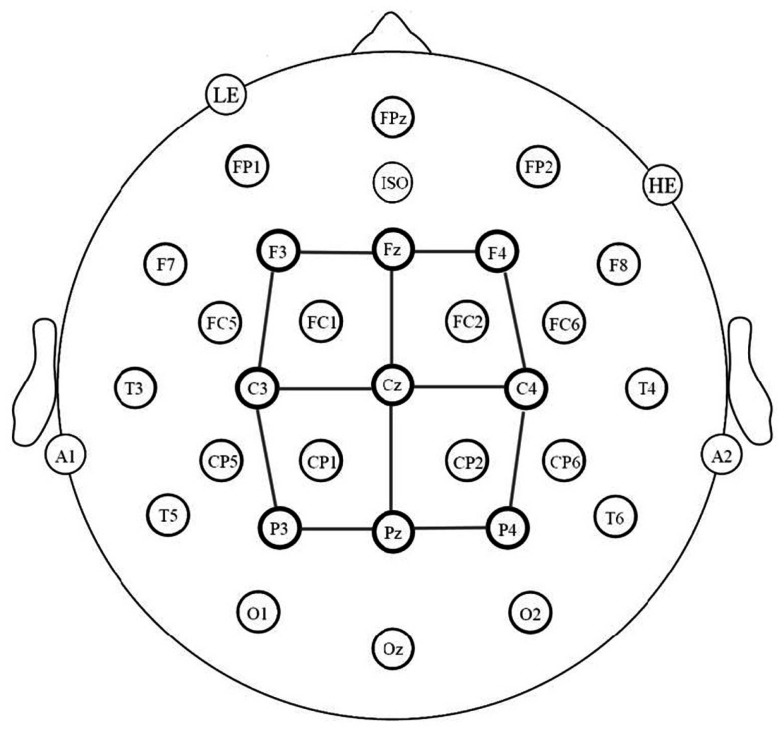
**Electrode montage and electrode sites used in the statistical analyses (connected by lines)**.

The electroencephalogram (EEG) was recorded using 32-channel caps (Electro-Cap International), in which tin electrodes were arranged following a revised standard International 10–20 system. Twelve electrodes were arranged in mirror images on each hemisphere: FP1/2, F3/4, F7/8, FC1/2, FC5/6, C3/4, T3/4, CP1/2, CP5/6, T5/6, P3/4, and O1/2 (see Figure 2). Five electrodes were located at midline: FPz, Fz, Cz, Pz, Oz. An electrode was placed below the left eye (LE) and another next to the right eye (HE) to monitor for blinks and saccades. Two electrodes were placed behind the ears on the mastoid process: the left mastoid site (A1) was used as an online reference for all electrodes, and the right mastoid site (A2) was also recorded to evaluate differential mastoid activity. All electrodes were referenced to the left mastoid (A1). An electrolyte gel was used in all electrodes in order to establish contact between the skin and the electrode. Reference electrodes impedances were kept below 2 kΩ, scalp electrodes impedances below 5 kΩ and eye electrodes impedances below 10 kΩ. The EEG was amplified using an SA Bioamplifier (SA Instruments, San Diego, CA, USA) operating on a bandpass of 0.01 and 40 Hz. The digitizing computer continuously sampled the EEG at a rate of 200 Hz.

### Data analysis

Averaged ERPs were formed off-line from trials free of ocular and muscular artifact (9% of trials were rejected for artifact) and were lowpass filtered at 15 Hz. Data analysis involved a representative subset of the 29 scalp sites (see Figure 2). Average waveforms were calculated off-line for three levels of language (L1 vs. L2 vs. L3), three levels of posterior-anterior (posterior, central, anterior), and three levels of laterality (right, center, left). As shown in Figure 2, a single electrode provided data for each of the nine conditions formed by the combination of the two distributional factors (posterior-anterior × laterality). Mean peak latency of the N400 component was calculated between 300 and 600 ms. Additionally, mean amplitudes were calculated in two epochs: 150–250 ms, to capture differences in P2 amplitude, and 350–500 ms, to capture differences in mean N400 amplitude between L2 and L3 which have the same peak latency^3^. Separate repeated measures analyses of variance (ANOVAs) were used to analyze the data in each of these three epochs as well as the N400 peak latencies. The Geisser and Greenhouse (1959) correction was applied to all repeated measures with more than one degree of freedom in the numerator.

## Results

### Behavioral data

We analyzed accuracy in detecting the animal probe words used for the purposes of the semantic categorization task (24 animal probes per language for a total of 72 animal words). Participants detected more animal probe words in L1 (*M* = 22.4, SD = 1.34) compared with both L2 (*M* = 16.0, SD = 4.70) and L3 (*M* = 15.7, SD = 4.75). The difference between L1 and L2 was significant [*t*(17) = 6.59, *p* < 0.001], as was the difference between L1 and L3 [*t*(17) = 6.81, *p* < 0.001], but the difference between L2 and L3 was not significant [*t*(17) < 1].

### Visual inspection of ERPs

**Figure 3:**
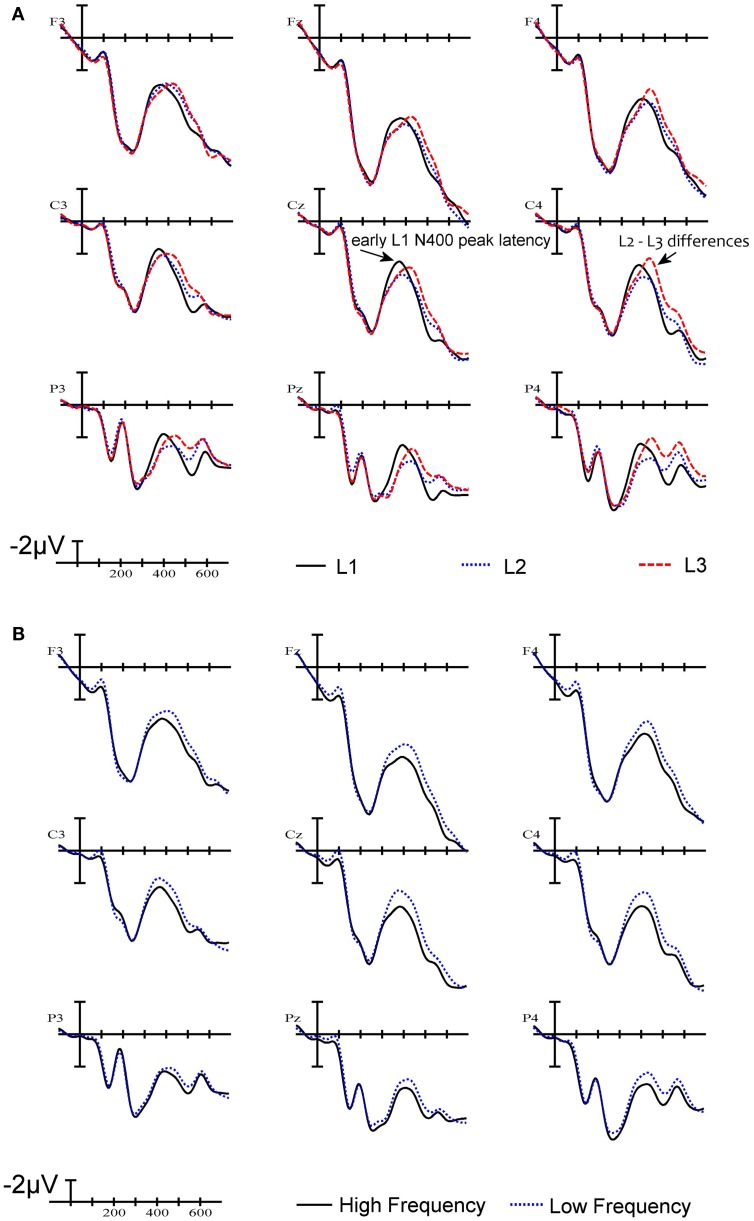
**Grand average waveforms**. **(A)** ERPs to words in L1 (French), L2 (English), and L3 (Spanish) at nine electrode sites. **(B)** ERPs to high frequency words and low frequency words. The *X*-axis indicates time from stimulus onset in milliseconds.

The ERPs time locked to stimulus onset are plotted in Figure 3A. As can be seen in Figure 3A, early in the waveforms there is a small negativity peaking around 100 ms (N1), followed by a considerable positivity peaking around 200 ms post-stimulus onset in frontal sites (P2). Up until about 200–250 ms, ERPs generated by the three languages are quite similar. At all sites there are differences on the negative-going waveform that start at about 250 ms and continue past 500 ms (N400). These negativities peak before 400 ms or after 400 ms depending on language. L2 and L3 tend to pattern together in the 300–500 ms epoch and are noticeably less negative-going than L1 at the beginning of this epoch. Here a clear distinction appears between L1 on the one hand and L2 and L3 on the other hand due to the early peaking of the L1 N400 component. This translates into a greater early negativity for the L1 words compared to words in L2 and L3, and a reversal of this pattern after 400 ms. Also notable is that in the later N400 window, L3 words elicit a larger negativity than L2 words.

### Analysis of mean amplitudes

For each epoch we first examined the main effect of language and its interaction with two electrode configuration factors: posterior-anterior and laterality, followed by planned pairwise comparisons between the three possible combinations of language: L1 vs. L2, L1 vs. L3, L2 vs. L3. We also compared the effects of language on ERP amplitudes with the effects of word frequency in all three languages. The statistical analyses of frequency effects are reported after the analyses of language effects.

### 150–250 ms epoch

There was no effect of language [*F*(2,34) < 0.1],and none of the interactions with language approached significance in this epoch. None of the pairwise comparisons between languages approached significance [L1 vs. L2: *F*(1,17) < 1; L1 vs. L3: *F*(1,17) < 1; L2 vs. L3: *F*(1,17) < 1].

### 350–500 ms epoch

Although there was no main effect of language in this epoch [*F*(2,34) = 1.7], there was a significant interaction between language and laterality [*F*(4,68) = 4.7, *p* < 0.009]. In pairwise comparisons this interaction was evident in the L2 vs. L3 comparison where L3 waveforms were significantly more negative-going than L2 waveforms at right hemisphere sites [language × laterality: *F*(2,34) = 4.4, *p* < 0.02]. The pairwise comparisons between L1 and L2, and L1 and L3 were not significant and did not interact with any of the distributional variables [all *F*s < 1 except for L1 vs. L3: *F*(1,17) = 3.1].

### N400 peak latency analysis

We calculated the peak latency in milliseconds of the N400 component at our nine electrode sites for each language. Over all nine electrodes mean peak latency for the N400 to L1 items was 393 ms while mean peak latency for L2 and L3 items was 430 and 432 ms respectively. The analysis showed a main effect of language [*F*(2,34) = 12.5, *p* < 0.001]. Pairwise comparisons between L1 and L2 showed that the L1 N400 peaked significantly earlier than the L2 N400 [*F*(1,17) = 13.6, *p* < 0.002]. The same pattern was found comparing L1 and L3 peak latency, with L1 peaking significantly earlier than L3 [*F*(1,17) = 19.6, *p* < 0.001]. However, there was no significant difference between L2 and L3 peak latency (*F* < 1).

### Word frequency analysis

Effects of word frequency were analyzed by dividing the critical words into two equal groups corresponding to the highest and the lowest frequency words within each language. Visual inspection of the waveforms revealed that word frequency modulated mean amplitude of the N400 component (see Figure 3B). An ANOVA of mean amplitude in the 350–500 ms epoch, with frequency and language as main factors, revealed a main effect of frequency [F(1,17) = 8.4, *p* < 0.01] and no interaction between frequency and language (*F* < 1). The main effect of word frequency is shown in Figure 3B. The pattern of word frequency effects follows the typical pattern reported in the literature, whereby high frequency words generate smaller N400 amplitudes than low frequency words (e.g., Van Petten and Kutas, 1990).

**Figure 4:**
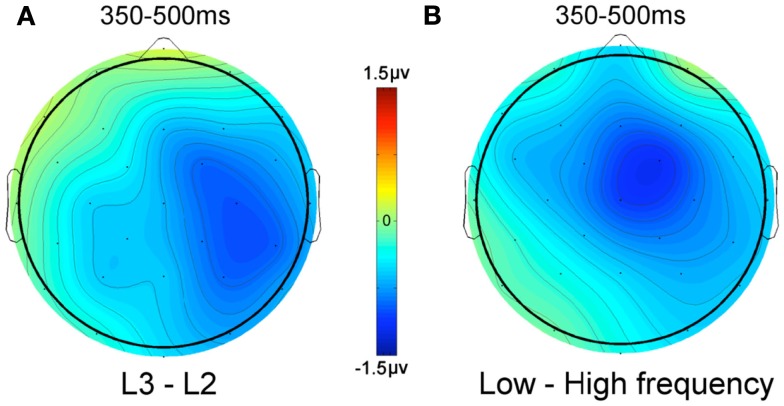
**Voltage difference maps**. **(A)** L3 words minus L2 words, **(B)** low frequency words minus high frequency words.

A comparison of the effects of word frequency and the difference between L2 and L3 words on N400 amplitude is shown in Figure 4. This shows that the L2–L3 language effect is quite similar to the word frequency effect, both in terms of amplitude differences and topography. The only notable difference is that the language effect has a more right-posterior distribution.

## Discussion

Event-related potentials were recorded as French-English-Spanish trilinguals read mixed lists of words in their three languages and pressed a button whenever the stimulus was an animal name, independently of the language in which it was written. The ERPs generated by non-animal words that were non-cognates in all three languages were analyzed in order to examine processing differences across languages. The waveforms generated by words in all three languages patterned together until about 250 ms post-stimulus onset. Following this, L2 and L3 words were found to have similar peak N400 latencies that were delayed compared with the N400 peak latency to L1 words. Furthermore, L3 words generated larger negativities in the N400 window than L2 words, as revealed in the mean amplitude analysis between 350 and 500 ms. Differences in N400 amplitude between L1 on the one hand, and L2 and L3 on the other, were not examined given the difference in peak latency that was observed between these conditions.

The first important result of the present study is that the delay in peak latency of the N400 component, found by Midgley et al. (2009) in bilingual participants for L2 (see also Ardal et al., 1990), was observed for both L2 and L3 in our trilingual participants. The N400 component generated by L2 and L3 words peaked together and did so later than the N400 generated by L1 words. The fact that a delay in the peak of the N400 was seen to the same extent for L2 and L3 words lends support to the hypothesis that it is the special status of the L1 that is driving this data pattern, and not differences in proficiency *per se*. Here, the key distinction is between the first language and those that are acquired later in life. Compared with first language acquisition, learning a new language later in life is generally characterized as being more laborious, effortful, and explicit, suggesting that different learning mechanisms are at play, even for pre-adolescent learners. Here we hypothesize that the simple fact that one language has already been learned should change the way that an L2 is learned, even perhaps for relatively young learners outside of the classroom. It is this qualitatively different type of learning process that would be the basis of different types of lexical and semantic connectivity thought to distinguish simultaneous (early) bilinguals from later learners of an L2 (Hernandez et al., 2005; Grainger et al., 2010).

It is important to distinguish this hypothesis from the hypothesized existence of a critical period for second language acquisition, according to which native-like acquisition can only be achieved up to a certain critical point of brain maturation, after which loss of plasticity is thought to be the major handicap for language acquisition (Lenneberg, 1976). Without debating the evidence for (e.g., Johnson and Newport, 1989) and against (e.g., Birdsong and Molis, 2001; Hakuta et al., 2003) such a position, we would simply point out that the period of consolidation of L1 acquisition would already impose a non-maturational “critical period” after which language acquisition will be affected by the prior acquisition of a different language (Hernandez et al., 2005). This does not exclude the possibility that maturational constraints might also play a role, and indeed both factors might be responsible for observed effects of the AoOA on processing a second language.

One other factor that might well influence L2 processing is the manner in which the later learned language is acquired. Differences are likely to emerge as a function of whether L2 is learned principally in a classroom setting compared with a more naturalistic learning environment. Even within the domain of more naturalistic language learning, we expect that one key distinction is whether or not the L2 is learned in reference to the L1 or in a more autonomous manner. Thus, a situation of prolonged immersion in L2 with little or no contact with the L1, as in the case of certain adopted children (e.g., Pallier et al., 2003), will likely create a context where L2 acquisition can proceed in a manner most similar to L1 acquisition. Obviously, the results of the present study do not allow us to evaluate the contribution of these different factors in driving the N400 peak latency difference between L1 and later acquired languages. Future work could address these important issues by examining language effects in trilinguals that have acquired their L2 in a more naturalistic immersive setting, and their L3 via more formal instruction. This could be usefully combined with a systematic study of language effects in bilinguals having acquired their L2 in different settings.

Finally, in the present study, L2 and L3 words were found to differ significantly in terms of mean N400 amplitude between 350 and 500 ms post-stimulus onset, with L3 words generating more negative-going waveforms than L2 words. This pattern is the opposite to what was predicted on the basis of the results of Midgley et al. (2009), where an increase in proficiency in L2 was found to result in more negative-going, and therefore more L1-like waveforms. Nevertheless, there was still a difference in N400 peak latency for L1 and L2 in the more proficient L2 group of participants in the Midgley et al. (2009) study, which complicates any interpretation of differences in N400 amplitude. Furthermore, becoming more proficient in an L2 for a bilingual person might well involve mechanisms that are distinct from those that underlie proficiency differences in L2 and L3 in trilingual persons. Concerning mechanisms that might be driving the difference in N400 amplitude to L2 and L3 words in the present study, it is important to note that the pattern of N400 amplitude differences between L2 and L3 was quite similar to the pattern revealed in an analysis of word frequency effects (high frequency vs. low frequency) collapsed across all languages (Figure 4). This fits with the idea that it is differences in the amount of exposure to L2 and L3 words that is the key factor driving the differences in N400 amplitude generated by these words. Further research manipulating the level of proficiency in L2 and L3 in trilingual participants should help elucidate this and other complex issues related to the acquisition and use of more than one language. One obvious next step with respect to the present study would be to test trilingual participants for which L2 proficiency is closer to L1 proficiency and more clearly distinct from L3 proficiency. This would provide a stronger test of the hypothesized special status of a language that is acquired first compared with later acquired languages. Furthermore, in future work it will be important to test for language effects in trilinguals using lists of words blocked by language, since this will help determine the extent to which differences across languages might be due to differences in the ability to control for cross-language interference.

In conclusion, we found a pattern of differences in the ERP waveforms that separated L1 words from both L2 and L3 words in terms of peak N400 latency. This was taken to reflect differences in the way a first language is acquired compared with later acquired languages, which have a long-lasting influence on the processing of words in these languages. Differences in the ERP waveforms thought to be related to differences in processing fluency in the L2 and L3 were found in the mean amplitudes of the N400. We would argue that ERPs provide a sensitive diagnostic tool for investigating word comprehension in multilinguals, and that the specific case of trilingualism provides important additional leverage with respect to understanding fundamental differences between the processing of L1 and later acquired languages.

## Conflict of Interest Statement

The authors declare that the research was conducted in the absence of any commercial or financial relationships that could be construed as a potential conflict of interest.

## Footnotes

^1^See Ardal et al. (1990) for an earlier report of similar findings, and Proverbio et al. (2004), and Proverbio et al. (2006) for similar research on sentence processing. A review of this earlier research can be found in Moreno et al. (2008).

^2^Here we do not examine the many other factors than could drive differences between the L1 and subsequently learned languages, such as the Age of Onset of Acquisition (AoOA) and the manner of acquisition (naturalistic vs. formal instruction) of the later acquired language. These are considered to be factors that can modulate an otherwise fundamental distinction between the acquisition of L1 and later acquired languages.

^3^Visual inspection of the waveforms in Figure 3A reveals a clearly earlier peaking N400 for L1 compared with both L2 and L3. This implies that any comparison of L1 amplitude with either L2 or L3 amplitude in the N400 time window would be contaminated by the latency shift.

## Appendix

### List of the words used in the ERP analyses.

**Table:**

L1 French items			L2 English items			L Spanish items		
Achat	Dessous	Lessive	Always	Gift	Puddle	Actitud	Espejo	Piel
Ailleurs	Devoir	Levure	Ashtray	Glass	Pumpkin	Ala	Esposa	Playa
Amer	Dimanche	Lisse	Back	Ground	Rain	Alegria	Esquina	Potro
Assez	Doigt	Moine	Bean	Handrail	Right	Alma	Estrecho	Puerta
Aussi	Douceur	Mou	Bedroom	Happy	Sailboat	Amargo	Fecha	Punto
Avoine	Endormi	Muet	Better	Hazelnut	Sailor	Amenaza	Gasto	Rama
Bain	Enfer	Œil	Blind	House	Scream	Amigo	Gente	Reino
Batteur	Ensuite	Œuf	Broom	Hunger	Seated	Ancla	Golpe	Reloj
Berceuse	Eescalier	Orage	Burden	Joiner	Sheet	Arbol	Granja	Rencor
Beurre	Espoir	Ordure	Carriage	Joke	Short	Astilla	Gusto	Revista
Bienvenu	Façon	Plaisir	Ceiling	Keyboard	Sieve	Azucar	Hierba	Rojo
Bijou	Falaise	Pneu	Cherry	Kiss	Sight	Azul	Higo	Rueda
Bouclier	Farine	Poivron	Chest	Knee	Slight	Bailarin	Iglesia	Salvaje
Caillou	Faubourg	Pomme	Claw	Lawn	Stain	Borracho	Invitado	Sangre
Cannelle	Fer	Presque	Clog	Lawyer	Staple	Burbuja	Jefe	Seguro
Casque	Fier	Prochain	Cloth	Leek	Stick	Calor	Lagrima	Sensatez
Chaleur	Fleur	Puits	Clothes	Link	Straight	Camino	Lavada	Silla
Chemise	Foi	Rayure	Copper	Lock	Swarm	Campana	Medida	Suero
Chou	Fou	Rhume	Crowd	Loud	Sweet	Carne	Mejilla	Tanteo
Cierge	Frayeur	Sapin	Dawn	Luck	Target	Cena	Mentira	Tarjeta
Citron	Fromage	Serment	Dish	Mean	Thief	Cepillo	Miedo	Tejido
Collant	Froment	Sœur	Drink	Mind	Thing	Cimbra	Moreno	Traje
Colle	Goudron	Soie	Drop	Mourning	Thunder	Ciudad	Muelle	Vara
Comble	Goutte	Soin	Evening	Navel	Track	Clavo	Musgo	Verde
Conduite	Gueule	Sommet	Eyebrow	Needle	Waiter	Consejo	Ocupado	Vestido
Coude	Haleine	Tablier	Fall	Noise	Wardrobe	Creencia	Olor	Viaje
Coussin	Honte	Temps	Felt	Parsley	Warm	Cuerno	Oreja	Vida
Crainte	Jambe	Tonneau	Fight	Pencil	Weight	Derrota	Orgullo	Viento
Croix	Jaune	Trou	Flake	Pillow	Witch	Detalle	Partido	Viuda
Cru	Jeune	Volet	Frame	Pound	Woman	Diente	Pecado	Vuelo
Cuir	Jumeau		Gallows	Powerful		Edad	Perdido	
Demain	Jupe		Garlic	Pretty		Enfoque	Piedra	
